# Braking the system: the aryl hydrocarbon receptor controls monocytes in homeostasis

**DOI:** 10.1172/JCI199952

**Published:** 2025-12-01

**Authors:** Jessica E. Kenison, Francisco J. Quintana

**Affiliations:** 1 Ann Romney Center for Neurologic Diseases, Brigham and Women’s Hospital, Harvard Medical School, Boston, Massachusetts, USA.; 2 Broad Institute of MIT and Harvard, Cambridge, Massachusetts, USA.; 3 Gene Lay Institute of Immunology and Inflammation, Brigham and Women’s Hospital, Massachusetts General Hospital, Harvard Medical School, Boston, Massachusetts, USA.

## Abstract

The aryl hydrocarbon receptor (AhR) is increasingly recognized as a physiologic modulator of the immune response, a function that extends beyond its established role as a sensor for environmental xenobiotics. In a recent report published in the *JCI*, Cros et al. demonstrate that the AhR restrains tonic, microbiota-driven inflammatory cytokine production in monocytes. Through the combined use of murine models, human ex vivo systems, and the analysis of patient-derived data, Cros and coworkers established that the AhR limits stimulator of IFN gene–induced (STING-induced) proinflammatory signals. These findings define cell type–specific physiologic roles for the AhR in the regulation of innate immunity and underscore its potential as a therapeutic target for the treatment of inflammatory and autoimmune diseases.

The immune system is continuously exposed to diverse microbial and dietary stimulants, many of which interact with innate immune recognition receptors (1, 2). This low-level, or “tonic,” stimulation is important for immune maturation, regulation, and tissue homeostasis but, if left uncontrolled, can contribute to chronic inflammation or autoimmunity (3, 4). Recent studies have investigated mechanisms that regulate baseline inflammatory signaling and prevent inappropriate immune activation, but regulatory pathways participating in the cell type–specific control of tonic microbial sensing remain incompletely characterized. In a recent study, Cros et al. demonstrated a role for the aryl hydrocarbon receptor (AhR) in limiting monocyte proinflammatory responses to microbiota-derived cues, providing insight into the molecular networks that maintain immune balance (5).

The AhR was first identified as a sensor for environmental pollutants such as dioxins and polychlorinated biphenyls (6, 7). However, accumulating evidence demonstrates that the AhR acts as a physiological regulator of the immune system, integrating signals from endogenous, microbial, and dietary ligands (4, 8, 9). These AhR agonists induce a broad range of immunologic effects depending on ligand type, cell type, and tissue microenvironment, with consequences for both innate and adaptive immunity (4, 10, 11).

## The AhR limits monocyte inflammatory responses to microbiota

**Figure 1:**
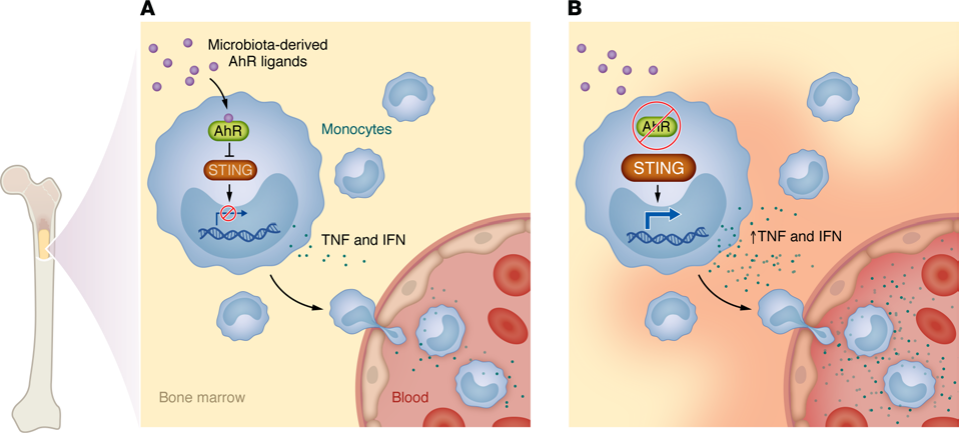
The AhR functions as a homeostatic brake on monocyte responses to microbiota. (**A**) The AhR restrains proinflammatory cytokine output by suppressing STING signaling, a function that maintains systemic immune balance. (**B**) Cros et al. reported that AhR deficiency in monocytes produced a proinflammatory response to tonic sensing of microbiota-derived signals (5).

In this issue of the *Journal*, Cros et al. used conditional genetic deletion to assess the AhR’s function in murine monocytes (5). They report that AhR deficiency in monocytes, but not in macrophages, resulted in increased spontaneous systemic inflammation, as evidenced by the upregulation of type I IFN-stimulated genes and increases in plasma TNF and chemokine levels (Figure 1) (5). Further analyses established that these proinflammatory effects of AhR inactivation in monocytes were dependent on microbiota-derived signals and the stimulator of IFN gene (STING) pathway, which is involved in cytosolic DNA sensing (12). Indeed, treating AhR-deficient mice with broad-spectrum antibiotics reversed this proinflammatory response, as did genetic deletion of STING, highlighting the importance of tonic microbiota sensing as a driver of immune dysregulation in the absence of AhR signaling (5).

Complementary ex vivo experiments in primary human monocytes revealed that AhR pharmacologic inhibition led to elevated cytokine production, recapitulating the findings in mice. In addition, the analysis of monocytes in systemic juvenile idiopathic arthritis (sJIA), a condition characterized by systemic inflammation, revealed both suppressed AhR activity and increased proinflammatory gene signatures, supporting the relevance of the AhR for the regulation of monocytes in human diseases (5).

This study adds to a growing body of work on the function of the AhR in immune homeostasis. The AhR has been implicated in modulating both innate and adaptive responses, for example by suppressing NF-κB and STING signaling, promoting Treg function, and maintaining barrier integrity (4, 11, 13, 14). Cros et al. suggest that monocyte-specific AhR activity serves to limit proinflammatory cytokine responses to persistent microbial stimulation. Notably, this role is not mirrored in macrophages, suggesting differences in developmental origin, chromatin landscape, or environmental exposure as potential determinants of AhR function (5). Further mechanistic studies are required to define the basis for these cell type–specific effects.

## Connecting the AhR to inflammatory and autoimmune disease biology

In addition to its homeostatic functions, the AhR has been identified as a candidate therapeutic target in multiple autoimmune diseases. Indeed, the therapeutic relevance of targeting the AhR has been underscored by the recent clinical approval of tapinarof, a selective AhR agonist, for the treatment of psoriasis (15, 16). Several ongoing clinical trials are evaluating AhR agonists for the treatment of other inflammatory and autoimmune diseases, including inflammatory bowel disease (17). The findings of Cros et al. are thus highly relevant for our understanding of the pathology and treatment of multiple systemic conditions, including rheumatoid arthritis, lupus, and multiple sclerosis, in which targeting the AhR may show therapeutic promise (18–20).

The environmental and metabolic contexts in which AhR operates add further dimensions to its biology. Dietary metabolites, microbial products, and endogenous host ligands have all been shown to influence AhR activity and thus immune regulation (4, 21, 22). These findings raise the possibility of modulating immune homeostasis through microbiome-targeted strategies or engineered probiotic approaches as alternatives to synthetic AhR agonists (23–25).

In summary, Cros et al. demonstrated that the AhR restrains tonic, microbiota-driven, STING-dependent cytokine responses in monocytes, supporting a broader role for the AhR in the maintenance of immune homeostasis. Their results deepen our understanding of the complexity and specificity of innate immune regulation and highlight the translational opportunities and challenges that will shape the development of AhR-based therapies for autoimmune and inflammatory diseases.

## Version 1 12/01/2025

Electronic publication
